# The Longitudinal Contribution of Early Morphological Awareness Skills to Reading Fluency and Comprehension in Greek

**DOI:** 10.3389/fpsyg.2017.01793

**Published:** 2017-10-13

**Authors:** George Manolitsis, Ioannis Grigorakis, George K. Georgiou

**Affiliations:** ^1^Department of Preschool Education, University of Crete, Rethymno, Greece; ^2^Department of Educational Psychology, University of Alberta, Edmonton, AB, Canada

**Keywords:** morphological awareness, reading fluency, reading comprehension, vocabulary, phonological awareness, rapid automatized naming, Greek

## Abstract

The purpose of this longitudinal study was to examine the role of three morphological awareness (MA) skills (inflection, derivation, and compounding) in word reading fluency and reading comprehension in a relatively transparent orthography (Greek). Two hundred and fifteen (104 girls; *M*_age_ = 67.40 months, at kindergarten) Greek children were followed from kindergarten (K) to grade 2 (G2). In K and grade 1 (G1), they were tested on measures of MA (two inflectional, two derivational, and three compounding), letter knowledge, phonological awareness, rapid automatized naming (RAN), and general cognitive ability (vocabulary and non-verbal IQ). At the end of G1 and G2, they were also tested on word reading fluency and reading comprehension. The results of hierarchical regression analyses showed that the inflectional and derivational aspects of MA in K as well as all aspects of MA in G1 accounted for 2–5% of unique variance in reading comprehension. None of the MA skills predicted word reading fluency, after controlling for the effects of vocabulary and RAN. These findings suggest that the MA skills, even when assessed as early as in kindergarten, play a significant role in reading comprehension development.

## Introduction

To date, several studies have shown that morphological awareness (MA) (the awareness of morphemic structures of words and the ability to reflect on them) is an important predictor of reading ability (e.g., Carlisle, 2003; Reichle and Perfetti, 2003; Deacon and Kirby, 2004; McCutchen et al., 2009; Tibi and Kirby, 2017; see also Ruan et al., 2017, for a meta-analysis) surviving the statistical control of other key predictors of reading ability such as vocabulary, phonological awareness, rapid automatized naming (RAN), and orthographic knowledge (e.g., McBride-Chang et al., 2005; Roman et al., 2009; Deacon, 2012; Desrochers et al., 2017).

However, previous studies examining the relationship of MA with reading ability have some important limitations. First, most previous studies have only examined a limited number of MA skills^1^ (see Rispens et al., 2008; Apel et al., 2013; Tibi and Kirby, 2017, for a few exceptions) and, as a result, different studies have operationalized MA with different tasks. It remains unclear if different aspects of MA predict reading ability the same way. Second, most of the studies reporting a connection between MA and reading ability have administered tasks based either on the explicitness of the morphological information (analogy, judgment, production) (e.g., Carlisle, 1995; Roman et al., 2009) or the representational level of morphological processes (inflection, derivation, and compounding) (e.g., Casalis and Louis-Alexandre, 2000; Vaknin-Nusbaum et al., 2016). However, assessing both the representational level of morphological processes and the explicitness of morphological information (e.g., examining the inflectional awareness by an analogy and a production task) can give us clearer evidence about the distinct roles of morphological processes in reading development. Finally, because most previous studies have examined the role of MA in reading after children had received formal reading instruction, it is possible that the observed effects were confounded by the effects of earlier reading ability on MA (e.g., Deacon et al., 2013; Kruk and Bergman, 2013; Cheng et al., 2016). Thus, the purpose of this study was to examine the role of three MA skills (inflectional, derivational, and compounding) in reading fluency and comprehension in a sample of Greek children followed from kindergarten to grade 2.

### Morphological Awareness and Reading Development

A number of studies have shown that MA predicts reading ability, particularly beyond the initial phases of learning to read (e.g., Deacon and Kirby, 2004; Roman et al., 2009; Kirby et al., 2012; Kieffer et al., 2013; Kim et al., 2013; Deacon et al., 2014; Pittas and Nunes, 2014; Desrochers et al., 2017). Similar positive relationships have been reported in intervention studies of MA (see e.g., Bowers et al., 2010, for a meta-analysis). Even though the majority of studies on MA have been conducted in English, there is now ample evidence that MA is important for reading ability across a wide range of languages such as Arabic (Tibi and Kirby, 2017), Chinese (Ku and Anderson, 2003), Dutch (Rispens et al., 2008), French (Casalis and Louis-Alexandre, 2000), Greek (Rothou and Padeliadu, 2015), Hebrew (Vaknin-Nusbaum et al., 2016), Japanese (Muroya et al., 2017), and Korean (Cho et al., 2011). Nunes and Hatano (2004) further argued that despite the different orthographic characteristics of different languages, MA is important for reading across languages.

A significant contribution of MA to reading ability is implied in the propositions of most contemporary theories of reading development (e.g., Frith, 1980; Marsh et al., 1981; Ehri, 2005; Seymour, 2005). For example, theories of reading development have postulated that during the later phases of reading development there is a shift in the strategies used for word reading from phonological recoding to the employment of larger linguistic units represented by morphemes. Recently, Kirby and Bowers (2017) also proposed that MA predicts reading because of the central role of morphology in binding the semantic, phonological, and orthographic information of words and in regulating these connections and the quality of words’ representation. Indeed, several studies in English have reported a significant effect of MA on both reading accuracy (e.g., Mann and Singson, 2003; Deacon and Kirby, 2004) and fluency (e.g., Apel and Diehm, 2014; Desrochers et al., 2017). However, theories of reading development suffer from an anglocentric focus (Share, 2008) and what we would expect for English may not apply to other languages with a more transparent orthography (e.g., Finnish, Greek).

Interestingly, the two behavioral studies that have examined the role of MA in word reading accuracy (Rothou and Padeliadu, 2015) and fluency (Desrochers et al., 2017) in Greek as well as the one that examined the effects of MA in decoding speed and accuracy in Finnish (Müller and Brady, 2001) have concluded that MA is not important for word reading in these languages. A similar conclusion was reached by some eye-movement studies for the role of morphology on word reading speed in Finnish and Dutch (e.g., Bertram and Hyönä, 2003; Kuperman et al., 2009, 2010; however, see also Häikiö et al., 2011).

The few behavioral studies in transparent orthographies have either examined inflectional skills (Müller and Brady, 2001; Rothou and Padeliadu, 2015) or general MA by combining the inflectional and derivational scores (Desrochers et al., 2017), and, therefore, we do not know if other MA skills (e.g., compounding) predict word reading fluency in these orthographies. If Greek children rely on orthographic knowledge (the ability to form, store and access phonological representations) to read fluently (e.g., Georgiou et al., 2008) and the orthographic units can represent morphemes as well, then MA should predict reading fluency. In contrast, if Greek children read fluently by converting the letters to sounds more efficiently (this expectation is based on the psycholinguistic grain size theory; Ziegler and Goswami, 2005), then MA should not predict reading fluency.

The method of reading instruction may also explain the non-significant effects of MA in word reading in transparent orthographies. More specifically, children in Finland and Greece receive phonics instruction to learn to read (e.g., Aro, 2006; Protopapas, 2017). This may give an unfair advantage to phonological awareness over MA. Indeed, several studies in transparent orthographies (including Finnish and Greek) have shown that phonological awareness predicts word reading fluency in grades 1 and 2 (e.g., Georgiou et al., 2008; Puolakanaho et al., 2008; Papadopoulos et al., 2009; Ziegler et al., 2010; Caravolas et al., 2012).

In contrast to reading fluency, a significant contribution of MA to reading comprehension would be expected based on the fact that words, beyond their phonetic aspects, carry morphological information and morphemes disclose the meaning of words (e.g., DeFrancis, 1989; Levesque et al., 2017). According to the lexical quality hypothesis (Perfetti, 2007), reading comprehension can be benefited largely from the knowledge of the components of words. These components include knowledge about word forms (e.g., grammatical class) and meaning representations. Building on this theoretical account, Levesque et al. (2017) further proposed that children’s morphological skills may actively contribute to the analysis of morphologically complex words’ meaning in order to influence the understanding of text. In support of the theoretical links between MA and reading comprehension, several studies have shown that general measures of MA predict reading comprehension (e.g., Carlisle, 1995; Carlisle and Fleming, 2003; Nagy et al., 2006; Kirby et al., 2012; Kieffer et al., 2013; Deacon et al., 2014; Pittas and Nunes, 2014; Desrochers et al., 2017; Levesque et al., 2017).

In addition, there is some evidence for the contribution of specific MA skills to reading comprehension, although this evidence is derived mostly from studies with school-age children. For example, Casalis and Louis-Alexandre (2000) found that inflectional awareness in kindergarten predicts reading comprehension in grade 2. Deacon and Kirby (2004) also found that inflectional awareness in grade 2 predicts reading comprehension in grades 4 and 5, and that the effects remain significant even after controlling for the effects of prior reading comprehension. Similarly, cross-sectional (e.g., Carlisle, 2000; Vaknin-Nusbaum et al., 2016; Deacon et al., 2017) and longitudinal (e.g., Carlisle, 1995; Casalis and Louis-Alexandre, 2000) studies have reported significant effects of derivational awareness on reading comprehension. In contrast to inflectional and derivational awareness, little is known about the relationship of compounding awareness with reading comprehension. To our knowledge, only Nagy et al. (2003) examined the role of compounding awareness in reading comprehension in English and included children at risk for reading difficulties^2^.

Clearly, longitudinal studies that look into the different representational processes of MA and their contribution to word reading fluency and reading comprehension are currently missing. A number of studies have focused on the link between MA and word reading accuracy (e.g., Singson et al., 2000; Deacon and Kirby, 2004; Rothou and Padeliadu, 2015), but there is lack of studies on reading fluency, which is used as the main reading outcome in transparent orthographies in which reading accuracy reaches ceiling soon after children receive formal reading instruction (e.g., Seymour et al., 2003; Duncan et al., 2013). For example, several studies have shown that by grade 2 Greek children score above 90% in word reading accuracy^3^ (e.g., Nikolopoulos et al., 2006; Papadopoulos et al., 2009; Protopapas and Gerakaki, 2009; Sarris and Dimakos, 2015). In addition, we need more studies examining the role of different MA skills in reading comprehension.

### The Present Study

The purpose of the present study was to examine the role of three MA skills (inflectional, derivational, and compounding) in reading fluency and reading comprehension in a sample of Greek children followed from kindergarten to grade 2. Special attention was paid to the inclusion of a number of known predictors of reading ability (non-verbal IQ, vocabulary, phonological awareness, RAN; see Georgiou et al., 2008; Manolitsis et al., 2009; Caravolas et al., 2012; Hulme and Snowling, 2013) in order to provide a rather conservative test of the effects of MA skills on reading fluency and comprehension.

Our study aimed to answer the following two questions:

(1) Do skills representing different morphological processes (inflection, derivation, and compounding) in kindergarten and grade 1 predict reading fluency in grades 1 and 2? Based on the findings of previous studies (e.g., Müller and Brady, 2001; Desrochers et al., 2017) and in light of the method of reading instruction in Greece, we expected that none of the MA skills would predict word reading fluency, after controlling for the effects of letter knowledge, phonological awareness, and RAN.
(1) Do skills representing different morphological processes (inflection, derivation, and compounding) in kindergarten and grade 1 predict reading comprehension in grades 1 and 2? We hypothesized that all MA skills would predict reading comprehension in grade 2, but not in grade 1. This is because children’s performance in reading comprehension in grade 1 is heavily influenced by their decoding skills (children who do not decode cannot comprehend text) and we control for the effects of key predictors of decoding such as letter knowledge, phonological awareness, and RAN. Because most Greek children master decoding by grade 2 (e.g., Seymour et al., 2003; Papadopoulos et al., 2009), the influence of the literacy-related skills (letter knowledge, phonological awareness, and RAN) on reading comprehension should diminish, and this should allow other skills such as MA to make a significant contribution^4^.

### The Morphological Features of Greek Language

Greek is a morphologically rich language characterized as a fusional type of language (Ralli, 2005), which has more than one morpheme per word and inflectional morphemes may convey multiple grammatical, syntactic, and semantic information. Greek has been estimated to have 41 inflectional suffixes for nouns (Kalamboukis, 1995). Inflectional morphemes are representing the grammatical markers of words for denoting words’ gender, person/number, tense, aspect, case. For example, there are inflectional markers to denote three different genders (masculine, feminine, and neuter) and four different cases (nominative, genitive, accusative, and vocative) for nouns and adjectives, two different numbers for nouns, adjectives and verbs, eight tenses, two voices (active, medium-passive), and three different aspects (perfective, imperfective, and perfect) for verbs (Holton et al., 2004).

In addition, there is a variety of derivational morphemes (prefixes and suffixes), although less productive than inflectional morphemes, which interact with inflectional morphemes in order to create new words (nouns, verbs, adjectives, and adverbs) added in stems^5^ (see Ralli, 2003). Derivational morphemes may change word’s grammatical category (e.g., /χor-ós/ ‘dance’ “noun” → /χor-év-o/ ‘I am dancing’ “verb”), but not in every case (see for example the prefix /dia/ added in the verb /γráfo/ ‘write’, resulting in the new verb /diaγráfo/ ‘delete’ with a different meaning from the initial root verb) (Ralli, 2005).

The lexical compounding system in Greek, which is much richer than in languages such as English and French, is a productive system in which the stem (and not the word as in other languages) plays a decisive role in word formation (for a detailed description of the Greek compounding system see Ralli, 2013). Compounding processes interact actively with inflectional and derivational processes of the Greek morphology to create one-word compounds by including lexical elements (lexemes) which “may be realized as stems, or words” (Ralli, 2013, p. 10). Greek compounds in their vast majority include a stem as the first lexical element, which is a part of a word without its inflectional ending morpheme and a stem or word as the second element depending on whether the compound’s both inflectional ending and its stress is different or identical with the ending and the stress of the second element, respectively. The two lexical elements of Greek compounds are linked with a semantically empty vowel /o/^6^, which is the compound marker [e.g., /trel-o-kóritso/ mad girl < trel(os) + koríts(i)], that ensures the transition from the first to the second element in a compound formation (Ralli, 2003, 2005) and its absence [/aγriànθropos/ ‘wild man’ < áγri(os) + ánθropos] occurs rarely due mainly to phonological or morphological grounds (Dalalakis, 1996; Ralli, 2013). The one-word compounds in Greek are classified mainly into the following categories according to their lexical elements. The two basic categories are (a) those who combine two stems [e.g., /trapez-o-mádil-o/ ‘tablecloth’ < trapéz(i) ‘table’ + madíl(i) ‘handkerchief’] called as [stem-stem] type, (b) those who combine two stems of which the second one is followed by an inflectional suffix making the second element, which is stressed appropriately, to stand as a whole word^7^ [e.g., /katsik-o-kléftis/ ‘goat-thief’ < /katsík(a) ‘goat’ + kléft(is) ‘thief’] called as [stem-word] type. The two less frequent categories consist of (c) two full word forms with the first element of the compound to be an uninflected word (e.g., /eksóporta/ ‘outdoor’ < ékso ‘out’ + pórta ‘door’, /ksana-pézo/ ‘replay’ < ksaná ‘again’ + pézo ‘play’) called as [word-word] type and (d) an uninflected word with a stem [e.g., /katosèdono/ ‘bedsheet’ < káto ‘down’ + sedón(i) ‘sheet’] called as [word-stem] type. To sum up, Greek compounds have three basic characteristics: (a) compounds receive stress, which does not always concur with the stress of the constituent parts, (b) compounds’ meaning may not be derived from the meaning of the constituent parts and (c) compounds’ constituent parts are usually stems and not words with the first part to be always an uninflected item (Dalalakis, 1996; Ralli, 2005, 2013). Based on these morphological characteristics of Greek language (see more in Dalalakis, 1996; Ralli, 2003), one would expect MA to be important for reading development, even as early as in grades 1 and 2.

## Materials and Methods

### Participants

To recruit our participants we first sent letters of information to the parents of all children attending 10 kindergarten schools in Heraklion, a typical urban city in Greece. Two hundred twenty-nine children (117 males and 112 females; *M*_age_ = 67.26 months, *SD* = 3.38, at the first time of measurement) with parental consent participated in the study and were followed in grade 1 (*M*_age_ = 79.39 months, *SD* = 3.33) and in grade 2 (*M*_age_ = 91.40 months, *SD* = 3.34). The children were native speakers of Greek and none had a formal diagnosis of intellectual, emotional, or sensory difficulties. By grade 2, our sample consisted of 215 children. Fourteen children (6.1% of the initial sample) withdrew from the study because their families moved to a different district and could not be located. In order to examine if the performance of the children who withdrew from the study differed significantly from the rest of the children, we performed *t*-tests on all their kindergarten performance scores. None of the *t*-tests reached significance (all *p*s > 0.15). Thus, the final sample consisted of 215 children (111 males and 104 females; *M*_age_ = 67.40 months, *SD* = 3.33, at the first time of measurement).

Mother’s educational level was recorded for the purpose of a different project and it was as follows: 7.2% of the mothers attended only elementary school, 11.3% attended only junior high school, 43.3% obtained a high school degree, 17.5% obtained a college degree and 20.6% obtained a university degree. Based on the National Statistics of Greece (National Statistical Service of Greece, 2007), the mother’s educational level in our study was representative of that of urban regions in Greece.

### Measures

#### Non-verbal IQ

The Raven’s Coloured Progressive Matrices (Raven, 1956) was used as a measure of non-verbal IQ. The task was administered only in kindergarten and required participants to select one of six options that best completed a matrix with a part missing. A participant’s score was the total number correct. Cronbach’s alpha reliability has been reported to be 0.90 (Sideridis et al., 2016).

#### Vocabulary

The Peabody Picture Vocabulary Test-Revised (Dunn and Dunn, 1981), which was adapted into Greek by Simos et al. (2011), was administered only in kindergarten to assess vocabulary knowledge. Participants were shown four pictures and the examiner said a word to describe one of the four pictures. Participants were then asked to look at the quadrant of pictures and point to the one that represented the word provided by the examiner. A participant’s score was the total number correct, since no standardized scores in Greek population have been established yet. The Cronbach’s alpha reliability coefficient in our sample was 0.96.

#### Letter Knowledge

This task was administered only in kindergarten and required participants to provide the sound of each of the 24 uppercase and lowercase Greek letters. The letters were arranged randomly on an A4 paper. The maximum score was 48. Letter name knowledge was not assessed because children in Greece are not taught the letter names (e.g., *alpha, beta*) before the end of grade 1. The Cronbach’s alpha reliability coefficient in our sample was 0.94.

#### Rapid Automatized Naming (RAN)

Rapid automatized naming was assessed in kindergarten with an Object Naming task and in grade 1 with a Digit Naming task. Both tasks were adapted in Greek from the Comprehensive Test of Phonological Processing (Wagner et al., 1999). Children were asked to name as fast as possible six recurring pictures (*apple, cat, key, ball, hen*, and *tree*) or numbers (4, 7, 8, 5, 2, and 3) that were arranged in semi-random order in four rows of nine. Prior to time testing, children were asked to name the objects or digits in a practice trial to ensure familiarity with the stimuli. Children named the objects or digits twice (in the second card, the items were rearranged). A participant’s score in Object and Digit Naming was the number of items in the task (36) divided by the average time to name both cards. Wagner et al. (1999) reported test–retest reliability for Object and Digit Naming to be 0.87 and 0.91, respectively.

#### Phonological Awareness

Syllable deletion with words (administered in kindergarten) and phoneme deletion with non-words (administered in both kindergarten and grade 1) were used to assess phonological awareness. In the syllable deletion task (Manolitsis, 2000), children were presented with a two- or three-syllable word and then asked to delete the first syllable from it and say what was left (e.g., Say /lemoni/ [lemon] without saying /le/ is /moni/ [alone]). In all 10 items, the part of the word that remained after deleting a syllable was a real word. A participant’s score was the total number of correct items. No discontinuation rule was applied in this measure. The Cronbach’s alpha reliability coefficient in our sample was 0.94. In the phoneme deletion task (Porpodas, 2008), children were presented with a one-syllable pseudoword (2–4 phonemes) and then asked to remove a phoneme from it and say what was left. The position of the phoneme to be removed varied across the 24 items: in 13 items it involved the initial phoneme (e.g., Say /lo/ without saying /l/ is o) and in 11 items it involved the final phoneme (e.g., Say /las/ without saying /s/ is la). A participant’s score was the total number correct. A discontinuation rule of three consecutive errors was applied in this measure. The Cronbach’s alpha reliability coefficient in our sample was 0.95 in kindergarten and 0.91 in grade 1.

#### Morphological Awareness

Seven tasks (two for derivational morphology, two for inflectional morphology, and three for compounding morphology), adapted into Greek by the second author from similar tasks in English and other languages (see below for more information), were given orally^8^ to the participants in kindergarten and grade 1 (see Appendix). A participant’s score in all tasks was the total number correct. No discontinuation rule was applied in these measures.

##### Word analogy

The word analogy task was adapted into Greek from Nunes et al. (1997) and was used as a measure of inflectional and derivational morphology. Children were asked to identify a morphological relationship between one pair of words and apply the same relationship to complete a second pair of words. It consisted of two conditions, inflectional and derivational, and it was preceded by four practice items (two for each condition). The inflectional condition (e.g., “walk-walked”, “help-…”) consisted of 10 items with several transformations of noun/inflection and verb/inflection. In five items, the stem of inflected word types remained unchanged [e.g., /γiatrós/ : /γiatrí/ :: /aetós/ : (/aetí/), for an English example ‘doctor’ : ‘doctors’ :: ‘eagle’ – (‘eagles’)] and in the other five items there was a change of the stem [e.g., /éklepsa/ : /klévo :: /éγrapsa/ : (/γráfo/), for an English example ‘stole’ : ‘steal’ :: ‘wrote’ : (write”)] that was based exclusively on morphological rules and not on phonological similarities. The derivational condition (e.g., “high: height”:: “deep : …..”) consisted also of 10 items with several transformations: transforming a base word into a derived word adding a suffix or a prefix and transforming a derived word into a base word. In five items, the stem of the base words remained unchanged after affixation [e.g., /ómorf-os/ : /omorf-iá/ :: /nóstim-os/ : (/nostim-iá/), for an English example (baker’ : ‘bakery’ :: ‘tasteful’ : (‘tasty’)] and in the other five items there was a change of the stem during transformation [e.g., /krív-o/ : /kri-ménos/ :: /váf-o/ : (/va-ménos/), for an English example “tolerate : tolerance” :: ‘fly’ : (‘flight’)]. The maximum score in each condition was 10. The order of the items within the two conditions was counterbalanced to avoid practice effects. The Cronbach’s alpha reliability coefficient in our sample was 0.86 in kindergarten and 0.85 in grade 1.

##### Production of inflected forms

This task was adapted into Greek from the “Production of Word Forms Test” (Carlisle and Nomanbhoy, 1993) and was used to assess children’s awareness of inflectional morphology. Children were provided with a target word and then asked to produce the correct inflected form of the target word in order to complete a grammatically, semantically, and morphologically correct sentence (e.g., “Dog. I saw ten …”). This task consisted of 20 items with several transformations of nominal and verbal inflection, and it was preceded by four practice items. Ten items required a transformation of nouns [e.g., /maθítries/. /I aðelfi mu íne (maθítria)/, for an English example “Students. My sister is a (student)”] or of verbs [e.g., /Kaθaríz-o/. /Ta peðiá tóra (kaθaríz-un)/, for an English example “We clean. My aunt now (cleans)”] without any change on their stem. Another 10 items required transformation of verb tenses with a change on their stem [e.g., /lúst-ika/. /Tóra eγü (lúz-ome)/, for an English example “Slept. Today, for a moment I (sleep)”]. The maximum score in this task was 20. The Cronbach’s alpha reliability coefficient in our sample was 0.78 in kindergarten and 0.73 in grade 1.

##### Manipulation of derived forms

This task was adapted into Greek from the “Test of Morphological Structure: Derivation – Decomposition” (Carlisle, 2000) and was used to assess children’s awareness of derivational morphology. It consisted of two subscales: the derivation subscale and the decomposition subscale and it was preceded by four practice items (two for each subscale). In the first one (derivation), which contained 10 items, children were provided with a target base word and then asked to produce the correct derived form by transforming the base word with suffixation in order to complete a short sentence [e.g., /psár-i/. /O θíos mu íne (psar-ás)/, for an English example “Farm. My uncle is a (farm-er)”]. The second subscale (decomposition), also consisting of 10 items, required children to make changes from a target derived word to a base word in order to complete a sentence [e.g., /ékt-os/. /Ta γatákia ítan (éksi)/, for an English example “Sixth. The kittens were (six)”]. In each subscale, half of the items required changes in the stem of the target base word or of the target derived word. The maximum score in this task was 20. The Cronbach’s alpha reliability coefficient in our sample was 0.80 in kindergarten and 0.79 in grade 1.

##### Compound word segmentation

This task was adapted into Greek from the “Analyses of Compound Words” task (Lyster, 2002) and the “Morphemic-Segmentation Test” (Casalis and Louis-Alexandre, 2000), and was used to assess children’s awareness of lexical compounding. The task consisted of 16 items and it was preceded by three practice items. Children were asked to pronounce separately the two lexical elements of a compound word (e.g., Can you guess which words made the big word “doorbell”?). We designed two conditions for this task. In half of the items, the lexical elements of the compounds were two uninflected stems [e.g., /riz-ó-γal-o/ ‘rice pudding’ < ríz(i) ‘rice’ + γál(a) ‘milk’] and in the other half the lexical elements consisted of an uninflected stem combined with a stem followed by an inflectional suffix which can stand as a whole word [e.g., /petr-o-pólemos/ ‘stone throwing’ < pétr(a) ‘stone’ + pólem(os) ‘war’]. Therefore, participants had to segment the two stems, making proper transformations in order to pronounce them as whole words [e.g., /alat-o-píper-o/ ‘salt and pepper’ → the child must answer: alát(i) ‘salt’ and piper(i) ‘pepper’]. The maximum score in this task was 16. The Cronbach’s alpha reliability coefficient in our sample was 0.88 in kindergarten and 0.87 in grade 1.

##### Compound word reversal

This task was adapted into Greek from the “Morpheme-Reversal Task” (Elbro, 1989) and it was used to assess children’s awareness of lexical compounding. The task consisted of 10 items and three practice items. Children were asked to reverse a compound word and to pronounce the new compound [e.g., /pikr-ó-γlikos/ ronounce the new compound /γlik-ó-pikros/, ‘bittersweet’ → ‘sweet bitter’ < pikr(ós) ‘bitter’ + γlik(ós) ‘sweet’]. This requires children to first recognize the two lexical elements of each compound word. Then children were asked to make a transformation in which the second element of the initial compound had to be put first and the first element second in order to create a new compound. Most of the resulting new compounds were not “legitimate” words in the Greek vocabulary, although all they convey a meaning that is easily understood by native speakers. All of the compounds consisted of two stems. The maximum score in this task was 10. The Cronbach’s alpha reliability coefficient in our sample was 0.87 in both kindergarten and grade 1.

##### Compound word production

This task is similar to the “Compound Structure Test” (Nagy et al., 2003) and it was initially developed in Greek by Tzakosta (2009) to assess children’s awareness of lexical compounding. The task consisted of 15 items and three practice items. Children were provided with two target words and were asked to pronounce the compound word that could be derived from the two words. In five items, the two target words were given in the same order as in the resulting compound (e.g., What word is formed from /çióni/ ‘snow’ and /ánθropos/ ‘man’? → /çionanθropos/ ‘snowman’), in five items the two target words were given in the opposite order from that of the resulting compound (e.g., “What would we call the /çimós/ ‘juice’ of the /domatàs/ ‘tomato’? → /domat-o-çimós/ ‘tomatojuice’), and in the last five items the two target words were creating a legitimate compound that does not exist in Greek (e.g., “What would we call the /trípa/ ‘hole’ of the /lemóni/ ‘lemon’? → /lemon-ó-tripa/ ‘lemonhole’). Children had to transform appropriately the target words into stems in order to pronounce successfully the resulting compound. The maximum score in this task was 15. The Cronbach’s alpha reliability coefficient in our sample was 0.81 in kindergarten and 0.85 in grade 1.

#### Reading Fluency

The word reading efficiency (WRE) from the Test of Word Reading Efficiency (Torgesen et al., 1999; see Georgiou et al., 2012a, for the Greek adaptation) was used in grades 1 and 2 to assess reading fluency. Children were asked to read a list of 104 real words as fast and accurately as possible within a 45-s time limit. A participant’s score was the total number of correctly read words within the specified time limit. Georgiou et al. (2012a) reported test–retest reliability for WRE to be 0.92 for elementary school children.

#### Reading Comprehension

To assess reading comprehension we used two standardized tests (one in each grade) that follow the same format and are group administered. The “Test of First Reading Comprehension” was designed by Porpodas (2008) and was used here in grade 1. The test consisted of 16 sentences of increasing difficulty and was preceded by four practice items. Children were asked to silently read each sentence and choose (by circling) among three alternative words the one that would correctly complete the sentence. In turn, the “Test for Detecting Reading Ability” was designed by Tafa (1995) and was used here in grade 2, because it is more suitable for children of this age. The test is timed (40 min are allowed for completion) and also uses a cloze format, whereby the children were asked to choose and underline from four alternative words the one that would correctly complete the sentence they had silently read. The test consisted of 42 sentences of increasing difficulty and was preceded by four practice items. The Cronbach’s alpha reliability coefficient in our sample was 0.89 in grade 1 and 0.91 in grade 2.

### Procedure

All participants were tested individually in a separate room in their schools during school hours by the second author and a graduate student who had experience in psychological testing. In kindergarten, testing lasted roughly one and a half hours and was completed in three sessions of 30 min each. In grade 1, testing lasted 1 h and was completed in three sessions. In grade 2, testing lasted 1 h and was completed in two sessions. In both grades 1 and 2, the reading comprehension task was administered in groups of 10–15 participants. Again, the administration of this task took place in a quiet room in school.

## Results

### Descriptive Analysis

Descriptive statistics for the measures used in the study are shown in **Table 1**. There were no missing data and all the analyses were performed with a full dataset of 215 participants. An examination of the distributional properties of the measures revealed that they were within acceptable levels (Kim, 2013). Before running any further analyses, the number of variables was reduced in order to limit task-specific variability. Particularly, we calculated composite scores for (a) inflectional, derivational and compounding morphology in kindergarten and grade 1, and (b) for phonological awareness in kindergarten. In kindergarten, the two phonological awareness tasks correlated 0.55 with each other. Likewise, the measures that made up each MA skill correlated higher than 0.50 with each other. In grade 1, the MA measures contributing to each composite score correlated 0.52 or higher with each other. In all instances, the composite scores were calculated by averaging the *z*-scores of the respective component measures.

**Table 1:** Descriptive statistics for all variables across grades.

	Kindergarten		Grade 1		Grade 2		Maximum score
				
Measures	*M*	*SD*	*M*	*SD*	*M*	SD	
Non-verbal IQ (raw scores)	16.78	3.65					36
Non-verbal IQ	97.46	12.94					
Vocabulary^a^	67.37	19.22					173
Letter-sound knowledge	21.62	16.49					48
Phonological awareness							
Syllable deletion	3.17	3.79					10
Phoneme deletion	1.78	4.01	12.61	7.82			24
Rapid automatized naming^b^							
Objects	0.67	0.16					–
Digits			1.41	0.35			–
Inflectional morphology							
Word analogy (inflections)	3.75	2.58	6.06	2.50			10
Production of inflected types	11.38	3.83	13.22	3.25			20
Derivational morphology							
Word analogy (derivations)	3.36	2.42	5.21	2.59			10
Manipulation of derived types	10.66	4.25	13.94	3.74			20
Compounding morphology							
Compounds segmentation	6.37	4.26	8.58	3.99			16
Compounds reversal	1.86	2.61	3.33	3.14			10
Compounds production	4.10	3.24	5.63	3.54			15
Reading comprehension			10.43	4.80	21.11	8.37	16^1^/42^2^
Word reading fluency			22.22	10.21	39.10	11.71	104


*^1^Maximum score for grade 1; ^2^maximum score for grade 2; ^a^raw scores; ^b^items per second.*

**Table 2** displays the zero-order correlations among all variables used in the following hierarchical regression analyses. The three MA skills in both measurement points correlated highly with each other cross-sectionally (*r*s ranged from 0.76 to 82) and longitudinally (*r*s ranged from 0.70 to 89). The three MA skills correlated moderately with word reading fluency (*r*s ranged from 0.32 to 0.47) and moderately to strongly with reading comprehension (*r*s ranged from 0.44 to 0.65).

**Table 2:** Correlations between the variables across grades.

Kindergarten	1	2	3	4	5	6	7	8	9	10	11	12	13	14	15	16	17	18
**Measures**	
(1) Non-verbal																		
(2) Vocabulary	0.40																	
(3) Mother’s Ed	0.14	0.30																
(4) Letter sound	0.29	0.50	0.42															
(5) RAN	0.22	0.37	0.20	0.47														
(6) PA	0.31	0.46	0.33	0.64	0.48													
(7) InflA	0.33	0.57	0.36	0.58	0.50	0.59												
(8) DerA	0.31	0.59	0.33	0.57	0.45	0.60	0.84											
(9) CompA	0.33	0.65	0.36	0.69	0.49	0.67	0.76	0.80										
**Grade 1**																		
(10) RAN	0.26	0.26	0.34	0.50	0.63	0.48	0.36	0.34	0.36									
(11) PA	0.29	0.45	0.33	0.59	0.39	0.52	0.54	0.52	0.58	0.44								
(12) InflA	0.31	0.62	0.38	0.56	0.38	0.54	0.75	0.71	0.74	0.29	0.61							
(13) DerA	0.32	0.63	0.38	0.61	0.38	0.61	0.70	0.78	0.79	0.31	0.61	0.80						
(14) CompA	0.30	0.64	0.36	0.68	0.42	0.66	0.74	0.75	0.89	0.39	0.66	0.79	0.82					
(15) WRE	0.27	0.33	0.33	0.57	0.54	0.54	0.40	0.36	0.47	0.74	0.59	0.39	0.43	0.52				
(16) Read Comp	0.32	0.48	0.42	0.53	0.39	0.43	0.44	0.44	0.52	0.41	0.56	0.56	0.57	0.55	0.51			
**Grade 2**																		
(17) WRE	0.19	0.33	0.31	0.53	0.51	0.43	0.34	0.32	0.41	0.67	0.52	0.35	0.37	0.43	0.83	0.48		
(18) Read Comp	0.30	0.61	0.44	0.64	0.43	0.58	0.59	0.61	0.65	0.48	0.60	0.64	0.68	0.69	0.61	0.60	0.58	


*Ed, education; RAN, rapid naming; InfA, inflectional awareness; DerA, derivational awareness; CompA, compounding awareness; PA, phonological awareness; WRE, word reading efficiency; ReadComp, reading comprehension; For Pearson *r*’s > 0.14, *p* < 0.05; for *r*’s > 0.18, *p* < 0.01; for *r*’s > 0.24, *p* < 0.001.*

### Predicting Word Reading Fluency and Reading Comprehension

Next, we performed hierarchical regression analyses to examine the contribution of MA skills to future reading fluency and comprehension. Specifically, we ran three separate models with the kindergarten predictors (**Table 3**) and three separate models with the grade 1 predictors (**Table 4**). In Model 1, we entered non-verbal IQ, vocabulary, and mother’s education as control variables at step 1 of the regression equation. At step 2, we entered as a block the literacy-related skills (letter-sound knowledge – only in kindergarten -, phonological awareness, and RAN). Finally, at step 3, we entered the MA skills one at a time. In Model 2, we repeated the steps 1 and 2 of Model 1 and entered the MA skills as a block at step 3 of the regression equation. Finally, in Model 3, we entered the control variables at step 1, the autoregressor (reading fluency or comprehension at an earlier point in time) at step 2, and the MA skills at step 3 of the regression equation (they were entered one at a time). **Tables 3**, **4** present the standardized beta coefficients, significance levels, and *R*^2^ changes in word reading fluency and reading comprehension.

**Table 3:** Results of hierarchical regression analyses with measures in kindergarten as predictors of reading skills in grades 1 and 2.

Kindergarten measures	Reading comprehension Grade 1		Reading comprehension Grade 2		Word reading fluency Grade 1		Word reading fluency Grade 2	
				
	β	Δ*R*^2^	β	Δ*R*^2^	β	Δ*R*^2^	β	*ΔR*^2^
**Model 1**								
(1) Control variables		0.35***		0.49***		0.21***		0.19***
Non-verbal IQ	0.11		0.06		0.13		0.06	
Vocabulary	0.37***		0.54***		0.27**		0.29***	
Mother’s education	0.29***		0.27***		0.23**		0.22**	
(2) Literacy-related skills		0.08***		0.11***		0.25***		0.21***
RAN	0.12		0.09		0.30***		0.32***	
Letter-sound knowledge	0.24**		0.24**		0.21***		0.32***	
Phonological awareness	0.08		0.18*		0.25***		0.04	
(3) Morphological awareness								
(a) Inflectional	0.01	0.00	0.17*	0.01*	-0.12	0.01	-0.15	0.01
(b) Derivational	0.03	0.00	0.21*	0.02*	-0.14	0.01	-0.13	0.01
(c) Compounding	0.08	0.00	0.10	0.00	-0.09	0.00	-0.15	0.01
**Model 2**								
(3) Morphological awareness		0.00		0.02*		0.01		0.01
Inflectional	-0.04		0.06		-0.04		-0.10	
Derivational	0.02		0.20*		-0.12		-0.02	
Compounding	0.09		-0.06		-0.02		-0.08	
**Model 3**								
(2) Autoregressor^1^			0.38***	0.09***			0.83***	0.52***
(3) Morphological awareness								
(a) Inflectional			0.27***	0.04***			0.01	0.00
(b) Derivational			0.30***	0.05***			-0.05	0.00
(c) Compounding			0.13	0.01			-0.07	0.00


*The beta coefficients reported are taken from the step in the regression equation in which the predictor variables were entered. ^1^The autoregressor for the outcome variable ‘reading comprehension’ and ‘word reading fluency’ in grade 2 was the corresponding measure in grade 1. ^∗^*p* < 0.05, ^∗∗^*p* < 0.01, ^∗∗∗^*p* < 0.001.*

**Table 4:** Results of hierarchical regression analyses with morphological awareness measures in grade 1 as predictors of reading skills in grade 2.

Measures	Reading comprehension Grade 2		Word reading fluency Grade 2	
		
	β	Δ*R*2	β	Δ*R*2
**Model 1**				
(1) Control variables (kindergarten)		0.49***		0.19***
Non-verbal IQ	0.06		0.06	
Vocabulary	0.54***		0.29***	
Mother’s education	0.27***		0.22**	
(2) Literacy-related skills (grade 1)		0.13***		0.37***
RAN	0.21***		0.57***	
Phonological Awareness	0.30***		0.23***	
(3) Morphological awareness (grade 1)				
(a) Inflectional	0.27***	0.03*	-0.06	0.00
(b) Derivational	0.30***	0.04*	-0.06	0.00
(c) Compounding	0.22**	0.02**	-0.07	0.00
**Model 2**				
(3) Morphological awareness (grade 1)		0.05***		0.00
Inflectional	0.17		-0.02	
Derivational	0.24**		-0.03	
Compounding	-0.03		-0.03	
**Model 3**				
(2) Autoregressor^1^	0.38***	0.09***		0.52***
(3) Morphological awareness (grade 1)				
(a) Inflectional	0.30***	0.04***	0.00	0.00
(b) Derivational	0.33***	0.05***	-0.06	0.00
(c) Compounding	0.30***	0.04***	-0.07	0.00


*The beta coefficients reported are taken from the step in the regression equation in which the predictor variables were entered; ^1^The autoregressor for the outcome variable ‘reading comprehension’ and ‘word reading fluency’ in grade 2 was the corresponding measure in grade 1. ^∗^*p* < 0.05, ^∗∗^*p* < 0.01, ^∗∗∗^*p* < 0.001.*

The results with the kindergarten predictors (see **Table 3**) showed first that none of the MA skills was a significant predictor of word reading fluency. Second, the awareness of inflectional and derivational morphology accounted for a small, but still significant, amount of variance (1 and 2%, respectively) in reading comprehension in grade 2, but not in grade 1. These effects remained significant, even after partialing out the effects of reading comprehension in grade 1 (autoregressor). Interestingly, when all three MA skills in kindergarten were entered as a block in the regression equation (see Model 2 of **Table 3**), they did not account for more unique variance in reading comprehension than when entered individually. In fact, the results of this analysis showed that only derivational morphology remained a significant predictor of reading comprehension.

The results with the grade 1 predictors (see **Table 4**) showed that none of the MA skills was a significant predictor of word reading fluency. In contrast, they accounted for a significant amount of variance in reading comprehension (2 to 4%; see Model 1 of **Table 4**). Similar to the findings of **Table 3**, the joint contribution of the three MA skills to reading comprehension did not substantially surpass their individual contribution (see Model 2 of **Table 4**). Again, only derivational morphology remained a significant predictor of reading comprehension when entered at the same step with the rest of the MA skills.

## Discussion

The present longitudinal study examined the role of specific MA processes (inflection, derivation, and lexical compounding) in word reading fluency and reading comprehension in Greek. Importantly, the longitudinal contribution of these skills was tested in the presence of other key predictors of reading ability such as general cognitive ability (non-verbal IQ and vocabulary), letter-sound knowledge, phonological awareness, RAN, and even of reading ability at an earlier point in time (autoregressor). Our results showed that the contribution of MA skills varies as a function of the reading outcome; being significant only when predicting reading comprehension.

In line with our hypothesis and with the findings of previous studies (e.g., Müller and Brady, 2001; Manolitsis, 2006; Kirby et al., 2012; Desrochers et al., 2017) MA skills did not predict word reading fluency in grades 1 and 2, after controlling for the effects of general cognitive ability, mother’s education, and literacy-related skills. An explanation might be that faster readers in grade 2 do not use morphological information for reading words efficiently. Häikiö et al. (2011), for example, showed that that the most advanced grade 2 Finnish readers read compound words through a whole word processing and not by using a morphologically based strategy. An alternative explanation might be that Greek children read fluently by speeding up the process of phonological recoding (what would be expected based on the psycholinguistic grain size theory; Ziegler and Goswami, 2005). Presumably, if decoding becomes fast enough, children should read individual words fluently. A similar argument was made by de Jong (2011) using discrete and serial RAN to predict discrete and serial word reading. Specifically, de Jong (2011) showed that in grades 1 and 2, serial RAN was more strongly related to serial and discrete word reading than discrete naming. This implies that children process individual letters in words serially (the same way they name the stimuli in serial RAN). However, this finding may also be an artifact of the method of teaching reading in Greece. Given that phonics is the preferred method of teaching reading, children would be more inclined to sound out the words than rely on larger orthographic (and morphological) units.

Our findings are in contrast to the findings of previous cross-sectional studies (Rispens et al., 2008; Apel et al., 2013) showing significant effects of derivational awareness on word reading fluency. Beyond the obvious difference in the design of the studies (our study was longitudinal), the contradictory findings may be due to the use of control variables in the present study and more specifically of RAN. To our knowledge, this is the first time the effects of RAN on word reading fluency have been controlled for before examining the role of MA skills in a longitudinal study (but see Müller and Brady, 2001; Roman et al., 2009; for cross-sectional studies). This is critical because RAN is one of the best (if not the best) predictors of word reading fluency in transparent orthographies (e.g., de Jong and van der Leij, 1999; Di Filippo et al., 2005; Landerl and Wimmer, 2008; Georgiou et al., 2012b) and because Object Naming requires semantic processing over and above access to phonological representations (e.g., Poulsen and Elbro, 2013; Liu and Georgiou, 2017).

In contrast to reading fluency, reading comprehension in grades 1 and 2 was predicted by the MA skills, even when they were measured in kindergarten and after controlling for the effects of other known predictors of reading comprehension such as vocabulary and phonological awareness. Although this is certainly not the first study to show that morphological skills predict reading comprehension (e.g., Nagy et al., 2003, 2006; Kirby et al., 2012; Deacon et al., 2014; Pittas and Nunes, 2014; Levesque et al., 2017), we have shown for the first time that both inflectional and derivational processes of MA in kindergarten contribute uniquely to reading comprehension 2 years later. The contribution of MA skills to reading comprehension in grade 2 doubled when these skills were assessed in grade 1 (whereas kindergarten measures of MA accounted for 1–2% of unique variance, grade 1 measures accounted for 2–4% of unique variance). The findings were also in line with our hypothesis that MA skills would predict reading comprehension in grade 2, but not in grade 1. This pattern of findings is similar to that of Carlisle (1995) showing no effects of MA from kindergarten to reading comprehension in grade 1, as well as to that of Casalis and Louis-Alexandre (2000) showing a unique effect of inflectional skills in kindergarten and of derivational skills in grade 1 on reading comprehension in grade 2.

Lexical compounding awareness was an important predictor of reading comprehension only when assessed in grade 1. It is possible that this aspect of MA, which reflects children’s ability to manipulate intensively compound’s words morphemic structure, is still underdeveloped in kindergarten and cannot contribute to future reading comprehension. The Greek compounding system is more complex than that of other European languages such as French and English (see Ralli, 2013) and this may be one of the reasons why lexical compounding contributes to reading comprehension in Greek; manipulating the structure of compound words gives children an opportunity to infer the meaning of Greek polysyllabic compound words.

Some limitations of the present study are worth mentioning. First, we used an existing measure to assess children’s word reading fluency. This has two important implications: First, because the words were read in isolation (not in context), this may have reduced the role of MA. Second, the first 40 or so words in the task are mostly one or two (stem + inflectional suffix) morpheme words. Again, this may have reduced our chances to find significant effects of MA on word reading fluency. Besides, it has been argued that morphological skills are important for word reading accuracy or fluency when the words to be read are multimorphemic or complex (e.g., Deacon et al., 2011; Carlisle and Kearnes, 2017). In fact, a recent study by Grigorakis and Manolitsis (2017) showed that MA in kindergarten was a significant predictor of compound words reading fluency in Greek, but not of derived words fluency (they are morphologically simpler than compounds^9^). Second, we administered only one type of reading comprehension measures (they both followed a cloze format). To the extent different types of reading comprehension measures are affected by different cognitive-linguistic skills (e.g., Cutting and Scarborough, 2006; Kendeou et al., 2012), our findings may not generalize to other reading comprehension outcomes. Third, because reading accuracy in Greek reaches ceiling soon after formal reading instruction (e.g., Seymour et al., 2003; Tafa and Manolitsis, 2008; Papadopoulos et al., 2009), we did not administer a reading accuracy task in our study, but only reading fluency tasks that require both accuracy and speed. Fourth, the weak effects of lexical compounding awareness – assessed in Kindergarten – on later reading skills may be due to the low performance of children on the morphological compounding tasks. Although the tasks measuring lexical compounding awareness are difficult for young children (e.g., Rispens et al., 2008; Manolitsis, 2017), we included multiple measures of this construct in order to capture even the most demanding parts of MA skills. Finally, we acknowledge that our classification of the MA tasks is not the only one. Deacon et al. (2008), for example, proposed a taxonomy of MA tasks that takes into account also the format of presentation (oral vs. written). Certainly, this warrants further investigation in a future study.

## Conclusion

Our findings add to a growing body of research in different languages showing that MA skills are important for reading comprehension development. This has some important psychoeducational implications. First, it implies that if we were to assess MA in kindergarten we would increase our chances to identify children at-risk for reading comprehension deficits. In fact, a couple of studies have shown that poor comprehenders experience difficulties in MA (Tong et al., 2011; Adlof and Catts, 2015). Second, our findings suggest that the “whole is not better than the part”; the joint contribution of all morphological aspects to reading comprehension was not any better than the contribution of derivational morphology alone. This suggest that if researchers cannot test children on all MA aspects, they may achieve the same goal by administering only derivational morphology tasks. Third, our findings suggest that early intervention of MA may have a positive effect on children’s future reading comprehension (see Lyster et al., 2016, for some promising findings). Finally, our findings suggest that Greek teachers should perhaps reconsider the way they teach reading by complementing phonics instruction with some MA activities (see Bowers and Bowers, 2017 and Manolitsis, 2017, on how this can be achieved). This would be more in accord with the findings of several studies showing that MA is important for reading comprehension as well as with the characteristics of Greek orthography.

## Ethics Statement

The study was approved by the Institute of Education of the Ministry of Education in Greece as well as the Research Ethics Committee of the Department of Preschool Education at the University of Crete. Written consent from parents and schools was also obtained prior to testing.

## Author Contributions

The three authors contributed to the present manuscript in the following way: GM supervised the data collection, ran the analyses, and wrote most of the paper. IG conceptualized the research project, developed the MA measures in Greek, collected the data in schools, and wrote parts of the paper. GG contributed to the interpretation of the findings and to the writing of the paper.

## Conflict of Interest Statement

The authors declare that the research was conducted in the absence of any commercial or financial relationships that could be construed as a potential conflict of interest.

## Footnotes

1 Morphological awareness skills vary according to the morphological processes they tap such as inflection, derivation, and lexical compounding, or according to the cognitive processes used to demonstrate a manipulation of words’ morphemic structure such as segmentation, production, judgment, analogical reasoning, identification of word relatives, and oral vs. written skills. In the present study, we were interested in different morphological awareness skills based on their morphological processes.

2 We did not consider the studies conducted in Chinese here (e.g., McBride-Chang et al., 2005; Li and Wu, 2015; Cheng et al., 2016; Su et al., 2017), because compounding awareness in Chinese is different from compounding awareness in alphabetic orthographies (Ruan et al., 2017).

3 A small lexicality effect would be expected based on (a) cross-linguistic evidence provided by Seymour et al. (2003) for shallow orthographies with simple syllable structure such as Greek and (b) the findings of Greek studies which reported lower achievement for reading non-words (accuracy rates ranged from 70 to 90%) than for reading real words (Protopapas et al., 2007; Georgiou et al., 2008, 2012b; Papadopoulos et al., 2009; Papadimitriou and Vlachos, 2014; Sarris and Dimakos, 2015).

4 A similar finding has been reported regarding the contribution of decoding and oral language skills to reading comprehension as part of the “Simple View of Reading” theoretical account. More specifically, studies have shown that as children grow older the contribution of decoding to reading comprehension decreases and the contribution of oral language skills increases (e.g., Adlof et al., 2006; Torppa et al., 2016).

5 In this study we worked on the assumption that there is no formal distinction between root and stem in Modern Greek language, because “the borders between roots and stems have been blurred, and there is no synchronically motivated distinction between the two” (Ralli, 2013, p. 8). Thus, we use here the term ‘stems’ instead of ‘roots’ to refer to the basic lexical units of Greek words, which cannot occur on their own and they need inflectional affixes in order to stand as independent words.

6 According to a less prominent view, the /o/ is a connecting morpheme (Dalalakis, 1996).

7 Those compounds who combine two stems of which the second one is followed by an inflectional suffix, which may make the second element to stand as a whole word, if it is stressed appropriately, belongs to the first category of the [stem-stem] type (e.g., /psar-ó-varka/ ‘fishing boat’ < /psári/ ‘fish’ + /várka/ ‘boat’).

8 In contrast to other studies that examined the role of morphology to reading development using written tasks (e.g., Häikiö et al., 2011; Kieffer et al., 2013; Vaknin-Nusbaum et al., 2016), we used oral tasks, in order to avoid the possible effects of reading ability on morphological awareness.

9 Greek compound words bear an inflectional suffix at the right lexical element, while they may involve simultaneously a lexical element which could be a derived item [e.g., /anem-o-ðarmèn-os/ ‘wind-swept’ < ánem(os) ‘wind’ + ðarmèn(os) ‘beaten’]. On the other hand, derived words in Greek usually has a simpler construction with the presence of a single stem and an affix (prefix or suffix) [e.g., /angelúði/ ‘litlle angel’ < angel(os) - úði, /anáksios/ ‘unworthy’ < an - áksios] and a shorter length than compounds (for differences among compounding, derivation and inflection in Greek, see Ralli, 2013).
