# Supplementary material for: The Longitudinal Contribution of Early Morphological Awareness Skills to Reading Fluency and Comprehension in Greek
Source: Front Psychol. 2017 Oct 13;8:1793. doi: 10.3389/fpsyg.2017.01793 (PMC5645533; doi:10.3389/fpsyg.2017.01793)
Supplement: Supplementary file 1 [file Appendix.pdf]

Appendix

Morphological Awareness Tasks

Word Analogy (inflection)

No	Original items	English translation[1]
1.	/ʝa'tri/- /ʝa'tros/: /ae'ti/[2]-…. (/ae'tos/)[3]	‘doctors’-‘doctor’ : ‘eagles’- …(‘eagle’)
2.	/perpa'to/-/pe'rpatisa/: /voi'tho/- …(/vo'ithisa/)	‘I walk’-‘I walked’ : ‘I help’-…(‘I helped’)
3.	/'ekopsa/- /'kovo/ : /'eγrapsa/ -…. (/'γrafo/)	‘I cut’- ‘I cut’ : ‘I wrote’-…(‘I write’)
4.	/'pino/- /'epina/ : /'vlepo/-…(/'evlepa/)	‘I drink’ – ‘I was drinking’ : ‘I see’-….(‘I was seeing’)
5.	/si'kono/ - /si'konome/ : /xte'nizo/-…. (/xte'nizome/)	‘I raise’ – ‘I raised’ : ‘I comb’-….(‘I am combed’)
6.	/'xano/- /'exasa/ : /'ðeno/-…(/'eðesa/)	‘I lose’ – ‘I lost’ : ‘ I tie’-…..(‘I tied’)
7.	/'dinome/- /'diθika/ : /ze'stenome/-….(/ze'staθika/)	‘I dress up’- ‘I dressed up’ : ‘I warm up’-…(I warmed up’)
8.	/a'rxizo/ - /a'rxizume/ : /ðu'levo/-….(/ðu'levume/)	‘I start’- ‘We start’: ‘I work’-…..(‘We work’)
9.	/θa proxo'riso/ - /proxo'ro/ : / θa oði'γiso/-…. (/oði'γo/)	‘I will move on’- ‘I move on’ : ‘I will drive’-…..(‘ I drive’)
10.	/'ðasos/- /'ðasi/ : /'laθos/-…(/'laθi/)	‘forest’-‘forests’: ‘mistake’-…..(‘mistakes’)

Note: [1] English translation corresponds roughly to the meaning of the Greek items and are not following always a corresponding morphological analogy rule, because of the morphological differences among languages; [2] Stress is indicated (across all tasks’ items in the Appendix) with the symbol ΄ before each stressed syllable and the stressed part of the syllable is always its vowel constituent (e.g., the word ‘ae'tos’ is pronounced as /aetós/); [3] Correct answers are the words in the parentheses.

Word Analogy (derivation)

No	Original items	English translation[1]
1.	/'skavo/ - /'skapsimo/ : /'trexo/ - ….(/'treksimo/)	‘dig’ – ‘digging’ : ‘run’ - ….(‘running’)
2.	/ko'lo/ - /kseko'lo/ : /ði'pso/ - ….(/kseði'pso/)	‘glue’ – ‘winkle’ : ‘thirst’ - …. **(**‘quench one's thirst’)
3.	/'omorfos/ - /omo'rfça/ : /'nostimos/ - ….(/nosti'mɲa/)	‘beautiful’ – ‘beauty’ : ‘delicious’ - …. (‘delicacy’)
4.	/'krivo/ - /kri'menos/ : /'vafo/ - ….(/va'menos/)	‘hide’ – ‘hidden’ : ‘paint’ - ….(‘painted’)
5.	/'spiti/ - /spi'taki/ : /'ðondi/ - ….(/ðo'ndaki/)	‘house’ – ‘small house’ : ‘ touth’ - …. (‘small tooth’)
6.	/ku'no/ - /'kunima/ : /xti'po/ - ….(/'xtipima/)	‘shake’ – ‘shaking’ : ‘ hit’ - ….(‘hitting’)
7.	/stamati'menos/ - /stama'to/ : /aγapi'menos/ - ….(/aγa'po/)	‘stopped’ – ‘stop’ : ‘ beloved’- ….(‘love’)
8.	/'fuska/ - /fu'skono/ : /kli'ði/ - ….(/kli'ðono/)	‘bubble’ – ‘bubbling’ : ‘key’ - ….(‘lock’)
9.	/'ftisimo/ - /'ftino/ : /'pçasimo/ - ….(/'pçano/)	‘spitting’ – ‘spit’ : ‘gripping’ - ….(‘grip’)
10.	/mi'kros/ - /mi'kreno/ : /xo'ndros/ - …./xo'ndreno/	‘short’ – ‘shorten’ : ‘thick’ - …. (‘thicken’)

Note: [1] English translation corresponds roughly to the meaning of the Greek items and are not following always a corresponding morphological analogy rule, because of the morphological differences among languages.

Production of inflected forms

No	Original items	English translation
1.	/kaθa'rizo/ : /ta pe'ðja 'tora/…..(/kaθa'rizun/)	“I clean up”: Children are ….. “cleaning up” now
2.	/γra'mis/ : /zo'γrafisa 'pente/…..(/γra'mes/)	“line” : I painted five….. “lines”.
3.	/γi'rizo/ : / 'avrio to pro'i o 'nikos/…..(/θa γi'risi/)	“I return” : Tomorrow morning Nikos….. “will return”.
4.	/'lustika/ : /'tora sto 'spiti e'γo/…..(/'luzome/)	“I bathed” : Now at home….. “I am bathing”.
5.	/ma'θitries/ : /i aðe'rfi mu 'ine/…..(/ma'θitria/)	“students” : My sister is a….. “student”
6.	/xo'revete/ : /xtes e'γo si'nexia/……(/'xoreva/)	“You are dancing” : Yesterday, …..“I was dancing” all the time.
7.	/ko'libise/ : /e'mis 'tora/…..(/koli'bame/)	“He swam” : We ….. “swim” now
8.	/ci'mame/ : /xtes se mɲa sti'γmi e'mis/…..(/cimi'θikame/)	“I sleep” : Yesterday ….. “we slept” for a while.
9.	/'epeses/ : /to mo'ro 'tora/…..(/'pefti)/	“You fell” : The baby is ….. “falling” now.
10.	/kseki'no/ : /me'ta a'po mɲa 'ora ta 'trena/….(/θa kseki'nisun/)	“start” : After an hour the trains….. “will start”
11.	/ku'razome/ : /e'mis 'tora/…../(kura'zomaste/)	“I’m tired” : We ….. “are tired” now
12.	/erγa'ton/ : /o 'kostas 'ine/…..(/e'rγatis/)	“workers” : Kostas is a….. “worker”
13.	/tro'mazun/ : /xtes se mɲa sti'γmi o 'takis/…..(/'tromakse/)	“They frighten” : Yesterday, Takis …. “was frightened" for a while
14.	/zo'γrafisan/ : /e'sis 'tora/……(/zoγra'fizete/)	“They painted” : You are ….. “painting” now

15.	ono'maton/ : /af'tos 'exi 'ð̲io/…..(/o'nomata/)	“names” : He has two….. “names”
16.	/'klei/ : /e'sis 'tora γia'ti/…..(/'klete/)?	“He cries” : why….. “are you crying” now?
17.	/'ftçaxnis/ : /xtes se 'mɲ̟a sti'γmi ta pe'ð̲ja 'to/…..(/'eftçaksan/)	“You make” : Yesterday, the children….. “made it” at a moment.
18.	/pa'pað̲es/ : /af'tos 'ine/…..(/pa'pas/)	“Priests.” : He is a….. “priest”.
19.	/'epeksa/ : /ta γa'tacia 'tora/……(/'pezun/)	“I played” : The kittens are now….. “playing”.
20.	/a'kute/ : /o 'nikos 'tora ð̲en/…..(/a'kui/)	“You listen” : Nikos doesn’t …… “listen” now.

Note: [1] English translation corresponds roughly to the meaning of the Greek items in order to retain the syntax according to the English norms.

Manipulation of derived forms

No	C	Original items	English translation[1]
1.	Der	/fo'ni/ : /o 'nikos 'tora/…..(/fo'nazi/)	“voice”: Nikos ….. “raises his voice” now.
2.	Der	/'sproxno/ : /to a'maksi 'θeli/…..(/'sproksimo/)	“I push” : The car needs to be…… “pushed”.
3.	Der	/ta'ksið̲i/ : /ta ka'ravʝa 'tora/…..(/taksi'ð̲evun/)	“travel” : The ships are ….. “ traveling” now.
4.	Der	/xroma'tizo/ : /i i'kones 'ine/…..(/xromati'stes/)	“color” : Images are…….”colored”.
5.	Der	/'scivo/ : /o 'sakis 'ine 'panda/…..(/sci'ftos/)	“I bend” : Sakis is always….. “bent”.
6.	Der	/'sið̲ero/ : /i mixa'ni 'ine/…..(/sið̲e'reɲ̟a/)	“iron” : The machine is…... “made of iron”
7.	Der	/'klino/ : /to ku'ti 'ine/…..(/kli'sto/)	“close” : The box is….. “closed”.
8.	Der	'/psari/ : /o 'θios mu 'ine/…..(/psa'ras/)	“fish” : My uncle is a….. “fisherman”.
9.	Der	/'zvino/ : /to ce'ri 'ine/…..(/zvi'sto/)	“extinguish” : The candle is….. “extinguished”
10.	Der	/'ksilo/ : /to kre'vati mu 'ine/…..(/'ksilino/)	“wood” : My bed is….. “wooden”
11.	D	/proe'ð̲rio/ : /o ba'bas mu 'ine/…..(/'proeð̲ros/)	“presidency” : My dad is….. “president”.
12.	D	/ð̲e'rmatino/ : /i 'zoni 'ftçaxtike a'po/…..(/'ð̲erma/)	“leather” : The belt was made of….. “ leather”
13.	D	/isixazo/ : /e'γo 'ime/…..(/'isixos/)	“I quieten” : I am….. “ quiet”.
14.	D	/'psisimo/ : /o pa'pus po'les fo'res/…..(/'psini/)	“baking” : The grandfather often…..”bakes”.
15.	D	/'xtistis/ : /o 'kostas 'tora/…..(/'xtizi/)	“builder” : Kostas now….. “builds”.
16.	D	/tre'xala/ : /e'mis 'tora/…..(/'trexume/)	“at a run” : We are ... “running” now.
17.	D	/'ektos/ : /ta γa'tacia pu 'metrisa 'itan/…..(/'eksi/)	“sixth” : The kittens I counted were….. “six”.
18.	D	/lemo'nað̲a/ : /'ftçaxtike a'po/…..(/le'moni/)	“lemonade” : Made of….. “lemon”.
19.	D	/ksexa'zmenos/ : /e'γo si'nexia/…..(/kse'xnao/)	“forgotten” : I keep…… “forgetting”.
20.	D	/traγuð̲i'stis/ : /'kaθe 'vrað̲I e'γo/…..(/traγu'ð̲ao/)	“singer” : Every night I….. “sing”.

Note: C = Condition; Der = Derivative condition; D = Decomposing condition; [1] English translation corresponds roughly to the meaning of the Greek items in order to retain the syntax according to the English norms.

Compound word segmentation

No	Items[1]	Lexical elements – Expected Answer
1.	/ri'zoγalo/	< /'riz(i)/ ‘rice’ + /'γal(a)/ ‘milk’
2.	/aγri'anθropos/	< /'aγri(os)/ ‘wild’ + /'anθrop(os)/ ‘man’
3.	/γalazo'prasino/	< /γa'laz(ʝo)/ ‘light blue’ + /'prasin(o)/ ‘green
4.	/trelo'koritso/	< /tre'l(o)/ ‘crazy’ + /ko'rits(i)/ ‘girl’
5.	/psarota'verna/	< /'psar(i)/ ‘fish’ + /ta'vern(a)/ ‘tavern’
6.	/me'ronixto/	< /'mer(a)/ ‘day’ + /'nixt(a)/ ‘night’
7.	/kse'roklað̲o/	< /kse'r(o)/ ‘dry’ + /kla'ð̲(i)/ ‘branch’
8.	/anavo'zvinume/	< /a'navum(e)/ ‘we light up’ + /'zvinum(e)/ ‘we turn off’
9.	/maxero'piruna/	< /ma'xer(ʝa)/ ‘knives’ + /pi'run(ia)/ ‘forks’
10.	/siγotraγu'ð̲o/	< /si'γa/ ‘slowly’ + /traγu'ð̲(o)/ ‘sing’
11.	/avγo'lemono/	< /av'γ(o)/ ‘egg’ + /le'mon(i)/ “lemon’
12.	/kuklo'θeatro/	< /'kukl(a)/ ‘doll’ + /'θeatr(o)/ ‘theater’
13.	/xarto'mandilo/	< /xa'rt(i)/ ‘paper’ + /ma'ndil(i)/ ‘handkerchief’
14.	/nero'potiro/	< /ne'r(o)/ ‘water’ + /po'tir(i)/ ‘glass’
15.	/kitrino'kokinos/	< /'kitrin(os)/ ‘yellow” + /'kokin(os)/ ‘red’
16.	/piʝeno'erxome/	< /pi'ʝen(o)/ ‘go’ + /'erxom(e)/ ‘come

Note: [1] English translations are not given for compound words, because there is not always a corresponding English word. For this reason we provided only the English translation of the lexical elements in the second column.

Compound word reversal

No	Items[1]	Lexical elements	Expected Answer
1.	/ç̲o'nonero/	< /'ç̲on(i) ‘snow’ + ne'r(o) ‘water’	> /ne'roç̲ono/
2.	/ksi'noγlikos/	< /ksi'n(os)/ ‘sour’ + /γli'k(os)/ ‘sweet’	> /γli'koksinos/
3.	/fi'ð̲oxorto/	< /'fið̲(i)/‘snake’ + /'xort(o)/ ‘grass’	> /xo'rtofið̲o/
4.	/ko'ndoxondri/	< /ko'nd(i)/ ‘short’ + /xo'ndr(i)/ ‘fat’	> /xo'ndrokondi/
5.	/ski'lospito/	< /'skil(os)/ ‘dog’ + /'spit(i)/ ‘house’	> /spi'toskilo/
6.	/a'spromavros/	< /'aspr(os)/‘white’ + /'mavr(os)/ ‘black’	> /mav'roaspros/
7.	/nero'luluð̲o/	< /ne'r(o)/ ‘water’ + /lu'luð̲(i)/ ‘flower’	>/lulu'ð̲onero/
8.	/spi'rtokuta/	< /'spirt(a)/‘matches’ + /ku't(ç̲a)/ ‘boxes’	> /ku'tospirta/
9.	/ste'nomakro/	< /ste'n(o)/ ‘narrow’ + /ma'kr(i)/ ‘long’	> /ma'krosteno/
10.	/cefa'loponos/	< ce'fal(i)/ ‘head’ + /'pon(os)/ ‘ache’	> /pono'cefalos/

Note: [1] English translations are not given for compound words (items and expected answers), because there is not always a corresponding English word. For this reason we provided only the English translation of the lexical elements in the second column.

Compound word production

No	Items *“What would we call …”*		Expected Answer[1]
1.	/ti 'fluð̲a tis pa'tatas/?	‘the peel of the potato’?”	> /pata'tofluð̲a/
2.	/'ena 'asximo pa'pi/?	‘an ugly duck’?”	> /asxi'mopapo/
3.	/tin 'tripa tu lemoni'u/?	‘the hole of the lemon’?”	> /lemo'notripa/
4.	/ton vi'vlion ti 'θiki/?	‘the books’ case’?”	> /vivlio'θiki/
5.	/'otan ci'mame stra'va/?	‘when I sleep awry’?”	> /stravoci'mame/
6.	/tis 'γatas to 'ð̲romo/?	‘the cat’s road;?”	> /γa'toð̲romo/
7.	/mɲ̲a 'pita a'po ko'rð̲oɲ̲a/?	‘a pie of cords’?	> /korð̲o'nopita/
8.	/tu sapu'niu ti 'fuska/?	‘the soap’s bubble’?”	> /sapu'nofuska/
9.	/to xi'mo tis do'matas/?	‘the juice of the tomato’?”	> /domatoxi'mo/
10.	/mɲ̲a sa'lata apo mo'livj̲a/?	‘a salad of pencils’?”	> /molivosa'lata/
11.	/'ena pe'ð̲i 'plusio/?	‘a rich child’?	> /plusi'opeð̲o/
12.	/'otan kri'fa ki'tazo/?	‘when I look secretly’?”	> /krifoki'tazo/
13.	/mɲ̲a xri'si 'miγa/?	‘a golden fly’?”	> /xri'somiγa/
14.	/ti fo'ʎ̲a tis γa'rið̲as/?	‘the nest of the shrimp’?”	> /γarið̲ofo'ʎ̲a/
15.	/'ena 'xoma 'kokino/?	‘a red soil’?”	> /koki'noxoma/

Note: [1] English translations are not given for compound words in the expected answers, because there is not always a corresponding English word. For this reason we provided only the English translation of the compound word’s description in the second column.
